# Evaluation of plasma muscle enzyme activity as an indicator of lesion characteristics and prognosis in horses undergoing celiotomy for acute gastrointestinal pain

**DOI:** 10.1186/1746-6148-10-S1-S7

**Published:** 2014-07-07

**Authors:** Clarisa R Krueger, Audrey Ruple-Czerniak, Eileen S Hackett

**Affiliations:** 1 Department of Clincial Sciences, Colorado State University, Fort Collins, CO, 80523, USA

## Abstract

**Background:**

In horses undergoing celiotomy for acute gastrointestinal pain, identification of variables correlating with lesion severity and location, and survival provide veterinarians and owners with information that aids in making informed decisions regarding appropriate treatment. Muscle enzyme activity is often increased in horses undergoing celiotomy for acute gastrointestinal pain and it is not known if muscle enzyme activity increase is specific to lesion type or impacts prognosis for survival. The objective of this study was to evaluate the relationship of pre-operative increase in muscle enzyme activities with intestinal lesion characteristics, specifically lesion location (large versus small intestine) and whether it was strangulating versus nonstrangulating, and case survival in horses undergoing celiotomy for acute gastrointestinal pain.

**Methods:**

Records of 241 horses undergoing exploratory laparotomy for colic were reviewed retrospectively. Evaluation of preoperative plasma aspartate aminotransferase (AST), creatine kinase (CK), sorbitol dehydrogenase (SDH), and gamma-glutamyltransferase (GGT) activities, fibrinogen and glucose concentrations, and hematocrit (HCT) and their association with gastrointestinal lesion characteristics and survival was performed.

**Results:**

Pre-operative increase in plasma CK and AST activity, and HCT and decrease in plasma bilirubin concentration were significantly associated with presence of lesions resulting in intestinal ischemia. Increase in plasma CK activity and HCT were significantly associated with a decreased probability of survival to hospital discharge. Plasma GGT and SDH activity, and glucose and fibrinogen concentration were not significantly associated with survival or severity of disease in multivariate analysis.

**Conclusions:**

Plasma muscle enyzme activity may be useful as a prognostic indicator in equine colic cases. Given that increases in plasma CK and AST activity were significantly associated with nonsurvival and the presence of intestinal ischemia, preoperative increase in these enzyme activities could assist in identification of disease severity and prognosis of horses undergoing celiotomy for acute gastrointestinal pain. Further study is indicated to elucidate the etiology of increased muscle enzyme activity in horses with surgical colic disease observed in this preliminary study.

## Background

In horses undergoing celiotomy for acute gastrointestinal pain, identification of variables correlating with lesion severity and location, and survival provide veterinarians and owners with information that can assist in determining appropriate therapy and deciding between conservative and surgical treatments. Variables including heart rate, packed cell volume, pain level, and blood glucose have been associated with severity of disease and case mortality in studies of horses with gastrointestinal insult [1-3]. Muscle enzyme activity is often increased in horses undergoing celiotomy for acute gastrointestinal pain [4]. Myonecrosis, confirmed histologically, has been reported in 3 horses with antemortem muscle enzyme elevation secondary to acute gastrointestinal disease [5]. It is not known if muscle enzyme activity increase is specific to lesion type or impacts prognosis for survival.

A retrospective study of horses undergoing celiotomy for acute gastrointestinal pain was performed to assess whether disturbances in plasma CK and AST activity could be linked to the severity of intestinal disease, or more specifically presence or absence of gross ischemia secondary to strangulating intestinal lesions, lesion location and survival. The objectives of this study were to evaluate the relationship between pre-operative increase in muscle enzyme activities and other clinicopathologic parameters and intestinal lesion characteristics, specifically lesion location (large versus small intestine) and whether it was strangulating versus nonstrangulating, as well as case survival in horses undergoing celiotomy for acute gastrointestinal pain. It was hypothesized that increased plasma CK and AST activity would be associated with increased severity of intestinal disease and decreased survival.

## Methods

A retrospective, computer-generated search of the medical record database was performed to identify adult horses, aged >1 year of age, from January 1, 2006 to December 31^st^, 2010 at the Colorado State University Veterinary Teaching Hospital that underwent celiotomy for acute gastrointestinal pain. This study was performed retrospectively using medical records as source material, therefore no ethical review was sought prior to study commencement in compliance with Colorado State University Institutional Animal Care and Use and Veterinary Teaching Hospital procedures. Preoperative complete blood count and blood chemistry panel analytes, lesion designation of large or small intestinal, gross evidence of intestinal ischemia secondary to strangulating lesions resulting in compression of intestinal bloodflow as indicated in the operative record, performance of intestinal resection, and short term survival were evaluated. Preoperative complete blood count blood samples collected into evacuated tubes with ethylenediaminetetraacetic anticoagulant and blood chemistry blood samples collected into evacuated tubes with sodium heparin anticoagulant were obtained by direct jugular venipuncture at the time of hospital admission and analysis was performed by the Colorado State University Veterinary Diagnostic Laboratory with identical analysis and standard of measurement throughout the study period. Short term survival was defined as survival to hospital discharge.

All continuous variables underwent Shapiro-Wilk analysis for normality. As variables were not normally distributed, continuous data were reported as median and interquartile range. Clinicopathologic variables were then categorized into those within or above laboratory reference ranges for regression analysis. Mixed effects logistic regression was used to evaluate associations between preoperative clinicopathologic values and outcomes of interest. Outcomes evaluated were survival to hospital discharge, and gastrointestinal lesion characteristics, including intestinal location, presence of intestinal ischemia due to strangulation, and performance of intestinal resection. Each outcome variable was analyzed using separate models. While CK and AST activity were the predictors of primary interest, to allow more complete analysis of both hepatic contribution to AST activity increase and other related factors associated with disease severity, clinicopathologic variables evaluated as potential predictors in the analyses included plasma CK activity, AST activity, glucose concentration, bilirubin concentration, gamma-glutamyltransferase (GGT) activity, sorbitol dehydrogenase (SDH) activity, hematocrit (HCT), and fibrinogen concentration. Univariable models were used to screen individual laboratory values. Laboratory values that passed initial variable screening (*P* ≤ 0.25) were included in multivariable model building. Final multivariable models were identified by use of a backward selection procedure with a critical α for retention of ≤ 0.05. After initial multivariable model building, previously excluded variables were reintroduced into the final model to ensure that the exclusion was appropriate. Confounding was identified by ≥ 20% change in parameter estimates when variables were individually removed from the multivariable models. First order interaction terms for main effects variables included in final models were evaluated. Odds ratios (OR) and 95% confidence intervals (95% CI) were calculated using the results of logistic regression models. Analysis was conducted using commercially available software (StataCorp, College Station, TX, USA).

## Results

Two-hundred forty one horses undergoing celiotomy for acute gastrointestinal pain were identified at the Colorado State University Veterinary Teaching Hospital in the predetermined time period. Of these, 161 survived to hospital discharge and 80 died or were euthanized during hospital treatment. Of the 80 non-surviving horses, 43 horses were subjected to humane euthanasia intra-operatively. Of these 43, 41 were euthanized based on poor prognosis due to the extent of non-viable intestine (34), gastrointestinal rupture (3), or extensive intra-abdominal adhesions (4); and 2 horses were euthanized due to a combination of poor prognosis and financial constraints associated with post-operative care. Thirty-seven horses were subjected to humane euthanasia post-operatively due to colic (9), endotoxemia (8), lack of improvement (5), post-operative ileus (3), peritonitis (2), anastomotic failure (2), colitis (2), incisional dehiscence (2), severe vaginal hemorrhage (1), neurologic dysfunction (1), gastric rupture (1), and laminitis (1).

Clinicopathologic variables at admission are described in Table 1, including variables associated with survival on univariate analysis which were AST and CK activity, glucose concentration, and HCT. Clinicopathologic variables included in the multivariable model built to investigate associations with the occurrence of death or euthanasia during hospitalization were plasma CK and AST activity, glucose concentration, SDH activity, and HCT. After the backward selection procedure was completed, only two variables, CK activity and HCT, were retained in the model (Table 2) and increases in either value were associated with a increased risk of death or euthanasia during hospitalization.

**Table 1:** Summary of selected clinicopathologic variables at the time of admission of horses with surgical colic. Results listed as median and [Interquartile range]. Following univariate analyses, significant differences (*P*<0.05) were detected between survival groups as indicated by *.

Parameter	Overall Median [IQR]	Survivors	Non-Survivors	Reference Range	Univariate *P*-value
Aspartate aminotransferase (IU/L)	345 [286 – 502]	332 [282 – 431]	391 [295 – 615]	185 – 375	*P*<0.01*

Bilirubin (μmol/L)	34 [26 – 48]	34 [26 – 50]	33 [24 – 46]	9 – 29	*P*=0.55

Creatine kinase (IU/L)	478 [272 – 1036]	421 [251 – 671]	826 [379 – 2866]	100 – 470	*P*<0.01*

Fibrinogen (g/L)	3 [2 – 4]	3 [2 – 4]	3 [2 – 4]	1 – 4	*P*=0.93

Gamma-glutamyltransferase (IU/L)	16 [12 – 26]	16 [12 – 26]	17 [12 – 26]	7 – 20	*P*=0.59

Glucose (mmol/L)	8.2 [6.7 – 10.8]	7.8 [6.6 – 10.0]	9.8 [7.4 – 13.4]	3.9 – 7.5	*P*=0.04*

HCT (%)	37 [33 – 43]	36 [32 – 40]	42 [36 – 50]	30 - 45	*P*<0.01*

Sorbitol dehydrogenase (IU/L)	9 [4 – 20]	8 [4 – 17]	11 [5 – 25]	0 – 12	*P*=0.07

**Table 2:** Results for the final multivariable logistic regression model describing clinicopathologic variables associated with death or euthanasia during hospitalization in horses undergoing surgical treatment of colic.

Variable	Categories	Odds Ratio	95% CI	*P*-value
Creatine kinase	> 470 IU/L ≤ 470 IU/L	2.4	1.3-4.3 Reference	<0.01

HCT	> 45% ≤ 45%	4.5	2.0-10.0 Reference	<0.01

Primary lesions were identified in the large intestine in 187 horses and within the small intestine in 54 horses. Clinicopathologic variables included in the multivariable model built to investigate associations with the location of the GI lesion were plasma CK and AST activity, glucose concentration, bilirubin concentration, GGT and SDH activity, and HCT. After the backward selection procedure was completed glucose, SDH, and CK were retained in the model (Table 3). Increase in SDH activity was associated with an increased probability of the lesion occuring in the large intestine while increases in glucose concentration or CK activity were associated with an increased probability of the lesion occuring in the small intestine.

**Table 3:** Results, including odds ratio (OR) and 95% Confidence Interval (95% CI), for the final multivariable logistic regression model describing clinicopathologic variables associated with the occurrence of lesions in the large intestine in horses undergoing surgical treatment of colic.

Variable	Categories	Multivariable model			Univariable screening		
		
		OR	95% CI	*P*-value	OR	95% CI	*P*-value
Glucose	>7.5mmol/L ≤7.5mmol/L	0.3	0.1-0.6 Reference	<0.01	0.3	0.1-0.6 Reference	<0.01

Sorbitol dehydrogenase	> 12 IU/L ≤ 12 IU/L	4.6	2.1-10.1 Reference	<0.01	3.2	1.5-6.8 Reference	<0.01

Creatine kinase	> 470 IU/L ≤ 470 IU/L	0.5	0.3-1.0 Reference	0.05	0.5	0.3-0.9 Reference	0.02

Lesions identified during celiotomy for acute gastrointestinal pain resulted in grossly visible strangulation and intestinal ischemia in 129 horses (non-strangulating in 112 horses) and required intestinal resection in 28 horses (no resection in 213). Clinicopathologic variables included in the multivariable models built to investigate associations of predictor variables with strangulating lesions and ischemia were plasma CK and AST activity, glucose concentration, bilirubin concentration, GGT and SDH activity, and HCT. After the backward selection procedure was completed for both models, CK activity, bilirubin concentration, AST activity, and HCT were retained in the model (Table 4). Increase in bilirubin was associated with a decreased probability of strangulation and ischemia while increased CK and AST activity, and HCT were associated with an increased probability of intestinal strangulation and ischemia. Clinicopathologic variables included in the multivariable model built to investigate associations with resection were plasma glucose concentration, GGT and SDH activity, and fibrinogen concentration. After the backward selection procedure was completed, no variables were retained in the model. None of the parameters evaluated were found to be associated with intestinal resection (*P*=0.47).

**Table 4:** Results, including odds ratio (OR) and 95% Confidence Interval (95% CI), for the final multivariable logistic regression model describing clinicopathologic variables associated with the a strangulating intestinal lesion in horses undergoing surgical treatment of colic.

Variable	Categories	Multivariable model			Univariable screening		
		
		OR	95% CI	*P*-value	OR	95% CI	*P*-value
Aspartate aminotransferase	>375 IU/L ≤375 IU/L	2.2	1.2-4.0 Reference	<0.01	2.8	1.5-5.0 Reference	<0.01

Bilirubin	> 29 μmol/L ≤ 29 μmol/L	0.3	0.2-0.6 Reference	<0.01	0.3	0.2-0.5 Reference	<0.01

Creatine kinase	> 470 IU/L ≤ 470 IU/L	2.6	1.4-4.9 Reference	<0.01	3.0	1.6-5.8 Reference	<0.01

HCT	> 45% ≤ 45%	5.9	1.9-17.9 Reference	<0.01	5.5	1.8-16.9 Reference	<0.01

## Discussion

This preliminary study is the first to specifically evaluate the association between increased plasma muscle enzyme activity and surgical colic lesions in horses. Increased CK and AST activity evident in horses with colic are frequently attributed to muscle trauma associated with lying down, thrashing, rolling, administration of intramuscular injections, and subsequent trailering to referral centers. Other authors have identified antemortem increase in CK and AST activity in three horses with colic that had corresponding post-mortem histologic evidence of myodegeneration, myofibril edema and necrosis in major muscle groups un-related to direct trauma [5]. Endotoxin-mediated injury of skeletal muscle and liver may occur in colic and may instead be responsible, in part, for the increase in plasma muscle enzyme activities, CK and AST, seen in horses with acute gastrointestinal disease. Studies in sheep have established circulating endotoxin as a primary mechanism of muscle injury and corresponding increases in plasma creatine kinase (CK) and aspartate aminotransferase (AST) activity [6]. Prospective measurement of endotoxin concentrations and effects on plasma muscle enzyme activity and skeletal muscle histology may be warranted and could further elucidate the etiology of such changes, especially if conducted in horses with naturally occuring gastrointestinal disease.

Specific isoenzyme activities of CK and AST were not evaluated in the present study, therefore the relative contribution of tissue injury is unknown. Several isoenzymes of CK exist, with the MM dimer primarily in skeletal muscle, MB and BB dimers in the gastrointestinal system and brain, and the BB dimer in the pancreas and kidney [7-9]. Aspartate aminotransferase has two isoenzymes, M-AST which is exclusively found in mitochondria and C-AST which originates from the cytoplasm and is mainly found in muscle, liver and myocardium [7,10]. There is no apparent tissue specificity for either AST isoenzyme and horses have a significantly greater cytosolic to mitochondrial enzyme ratio than other species [11]. Peak CK activity is expected within 4-6 hours after acute muscle injury, with a circulating plasma half-life of approximately 123 +/- 28 minutes [12]. Alternatively aspartate aminotransferase activity peaks in the blood approximately 12-24 hours after muscle or liver injury and has a half-life of approximately 7 to 10 days [13,14]. Due to a short half-life and rapid peak post-injury, it is likely that identification of increased CK activity represents tissue injury relevant to the gastrointestinal lesion present on hospital admission and measurable plasma activities will precede those of AST activity secondary to the same tissue injury etiology.

Endotoxemia, a common complication of gastrointestinal disease, has been identified in approximately 30 to 40 % of horses presenting for colic [15-18]. It is well documented that circulating bacterial toxins activate endothelial cells, monocytes and granulocytes in the peripheral blood system and Kupffer cells within the hepatic sinusoids [19]. Activated Kuppfer cells produce inflammatory cytokines which potentiate the inflammatory cascade and can result in hepatic injury that can contribute to elevations in AST and CK activities and other hepatic enzymes. A previous report evaulating plasma biochemical alterations in experimentally induced low-flow ischemia to the large colon found significant elevations in systemic and colonic venous CK and AST activities [20]. However, as multiple isoenzymes of both AST and CK exist, the limitation of identification of isoenzyme and thus, source of isoenzyme presents a confounding factor that contributes to elevations of these enzymes. Additionally, the lack of an early discriminating hepatic functional assay delays recognition of hepatic injury [21-23]. Although a recent report has evaluated serum alcohol dehydrogenase as a useful clinical parameter in detecting intestinal strangulation in acute abdominal cases, and is routinely used to assess hepatic dysfunction in humans, this spectrophotometric analysis is not widely available to most practitioners and requires specific analyzer capability [24]. Another commonly evaluated non-specific enzyme, lactate dehydrogenase was not evaluated in the current study as it is not a component of our diagnostic profile and has a multitude of iso-enzymes which limit clinical application [12].

Similar to the findings of the present study, a recent report also found significant increase in AST activity, without significant increase in GGT activity, in acute intestinal obstruction when compared to non-obstructed controls [24]. Serum GGT activity increase has been associated with right dorsal displacement of the colon which compresses the bile duct resulting in extrahepatic bile duct obstruction [25]. In the present study, the hepatic cytoplasmic enzyme SDH activity was found to be increased in horses with large intestinal lesions which may be attributed to endotoxin-mediated cellular injury or secondary to biliary outflow obstruction due to compression from large intestinal distension, impaction, displacement, or volvulus. Also in the present study, increased CK and AST activity, and not GGT or SDH activity, was associated with increased disease severity and decreased survival. These results imply that while multiple tissues may contribute to CK and AST activity increase, skeletal muscle injury may be the most significant contributor. The clinical course of intestinal ischemia is often rapid, and sufficient time may not exist for prominent liver damage to occur and subsequent release of associated transaminases [26]. Further, horses in the present study with increased plasma bilirubin concentration were less likely to have a strangulating intestinal lesion implying that in less serious intestinal disease, longer duration of disease prior to referral may allow suffient time for bilirubin concentration increase to reach statistical difference when compared with those horses with more a serious disease and rapid disease course.

A limitation of the present study is failure to quantify in each enrolled horse the degree of musculoskeletal stress, presence of intramuscular injections, and amount of visible or observed self-inflicted trauma associated with the gastrointestinal pain episode. Increased muscle enzyme activity could likely have been attributed to multiple factors resulting in musculoskeletal stress and injury. One suggested mechanisms to account for skeletal muscle injury, and subsequent increases in plasma muscle enzyme activity observed in the present study, includes direct membrane injury by endotoxins and increase in skeletal muscle TNFα and free-radical induced lipid peroxidation, which ultimately lead to cellular dysfunction [27,28]. Metabolic stress induced by the increase in circulating cytokines and endotoxins can lead to an initial hypermetabolic state that quickly exhausts and overwhelms the mitochondria [29]. This leads to depletion of ATP stores and failure of energy dependent regulatory mechanisms that result in cellular swelling and lysis [7]. Several animal sepsis models have documented a decrease in mitochondrial function and subsequent increase in enzymes in skeletal muscle [30,31]. Free radical induced skeletal muscle damage is also noted in reperfusion injuries in human studies, which contributed to prolonged morbidity and recovery [29,32,33]. In an experimental endotoxemia model, infusion of lipopolysaccharide into the peritoneal space of horses resulted in increased serum CK activity, suggesting a causal relationship between circulating endotoxins and CK enzyme activity [34].

Previous reports document a direct correlation between plasma endotoxin concentration and both lesion type and severity of colic in horses [18]. Though endotoxin was not routinely measured in the present study, increase in CK and AST activity and HCT were found to be significantly associated with an increased probability of a strangulating lesion and presence of ischemia. Also in the present study, hyperglycemia and increased HCT were significantly associated with nonsurvival on univariate analysis. Both hyperglycemia and increased PCV have previously been documented to correlate with lesion severity and survival [1,3]. Exposure to LPS has been shown to result in hyperglycemia in horses [35,36]. Increased glucose in endotoxemia or other hypermetabolic states is directly attributed to tissue insulin resistance and endocrine derangement and reflects more severely disrupted homeostatic mechanisms [29]. While not specifically elucidated, similar mechanisms could also be responsible for the hyperglycemia and increase in CK activity observed in the present study in horses with small intestinal disease.

Hemolysis has an additive effect on CK activity measurement when spectrophotometric methods of measurement are used, due to the red blood cell enzyme adenylate kinase. Most commercial CK activity kits employ adenosine monophosphate and/or diadenosine pentaphosphate as adenylate kinase inhibitors. In the case of massive hemolysis, hemoglobin concentration of the serum can be measured to correct the apparent CK activity. In most settings this increase is not clinically significant and exclusion of hemolysed specimens is unnecessary [37]. Presence of hemolysis was not specifically addressed in the present study.

Limitations of retrospective study include reliance on the medical record for case data. In the present study, all studied variables were available for each case. However, greater detail on specific musculoskeletal stressors was not available.Prospective studies allow purposeful recording of variables with predetermined interest, such as those used to estimate disease severity. Further, evaluation of longer term followup would allow collection of more complete information on recovery, relapse, death, development of new disease, and the relationship of muscle enzyme activity with survival time.

## Conclusions

Increases in plasma CK and AST activity were significantly associated with the presence of intestinal ischemia and plasma CK activity was also significantly associated with patient survival to hospital discharge, which suggests that enzyme activity may be useful pre-operative indicator for equine surgical colic cases. Prospective studies are necessary to further examine this relationship.

## Competing interests

The authors declare they have no competing interests.

## Authors' contributions

CK participated in the data analysis and draft of the manuscript, AR participated in data statistical analysis, and EH participated in the design of the study, data collection and analysis, and draft of manuscript. All authors read and approved the final manuscript.

## Declarations

Funding for this study, including publication costs, was provided by the Colorado State University Advances in Equine Health Fund. The authors acknowledge Dr. Lindsay Piotrowski who conceived of the study and participated in study design and data collection.

This article has been published as part of *BMC Veterinary Research* Volume 10 Supplement 1, 2014: Selected articles from the Eleventh International Equine Colic Research Symposium. The full contents of the supplement are available online at http://www.biomedcentral.com/bmcvetres/supplements/10/S1. Publication of this supplement has been funded by The British Equine Veterinary Association.
